# Scoliosis in Herlyn–Werner–Wunderlich Syndrome: A Case Report and Literature Review

**DOI:** 10.1097/MD.0000000000000185

**Published:** 2014-12-02

**Authors:** Zheng Li, Xin Yu, Jianxiong Shen, Jinqian Liang

**Affiliations:** From the Department of Orthopaedic Surgery (ZL, XY, JS, JL), Peking Union Medical College Hospital, Peking Union Medical College, Beijing, China.

## Abstract

Herlyn–Werner–Wunderlich syndrome (HWWS) is a congenital Müllerian duct anomaly characterized by uterine didelphys, obstructed hemivagina, and ipsilateral renal agenesis. Little is reported about spinal deformity associated with this syndrome.

This study presents a case of scoliosis occurring in the setting of HWWS and explores the possible association between the 2 diseases.

A previously unreported scoliosis in HWWS is described. The patient is a 12-year-old Chinese female with scoliosis that underwent a posterior correction at thoracic 5–thoracic 12 (T5–T12) levels, using the Moss-SI (Johnson & Johnson, American) spinal system. At 24-month follow-up, the patient was clinically pain free and well balanced. Plain radiographs showed solid spine fusion with no loss of deformity correction. Six months after scoliosis correction surgery, the patient went to our clinics for the treatment of HWWS. She was performed a vaginal septum resection and detected with pyocolpos. Her follow-up was symptom free at the fourth postoperative month. The prevalence of scoliosis among patients with HWWS was 8.57% that is much higher than the incidence of congential scoliosis among general population (1/1000).

To the best of our knowledge, this is the first report of HWWS with thoracic scoliosis. During surgery, surgeons and anesthesiologists must pay particular attention to the Müllerian duct anomaly and renal agenesis associated with HWWS. There is a potential association between congenital scoliosis and HWWS.

## INTRODUCTION

Herlyn–Werner–Wunderlich syndrome (HWWS) is a rare congenital Müllerian duct anomaly characterized by uterine didelphys, obstructed hemivagina, and ipsilateral renal agenesis, which is first reported in 1922.^1,2^ Although the true incidence of this syndrome remains unknown, the overall incidence of obstructive Müllerian duct anomalies can range from 0.1% to 3.8%.^3,4^ HWWS is usually discovered at puberty with nonspecific symptoms, such as increasing pelvic pain, dysmenorrheal, and palpable mass due to the associated hematocolpos or hematometra, which result from retained, longstanding menstrual flow in the obstructed vagina.^3–7^ The exact cause, pathogenesis, and embryologic origin of HWWS remain a subject of discussion.^8^ There are limited reports regarding the diagnosis and management of HWWS with its possible resultant congenial scoliosis. We here present a case of HWWS in a 12-year-old girl with unusual presentation—congenital scoliosis.

## CONSENT

Written informed consent was obtained from the patient's parents on behalf of the child for publication of this case report and any accompanying images. A copy of the written consent is available for review by the Editor of this journal.

## CASE REPORT

**FIGURE 1:**
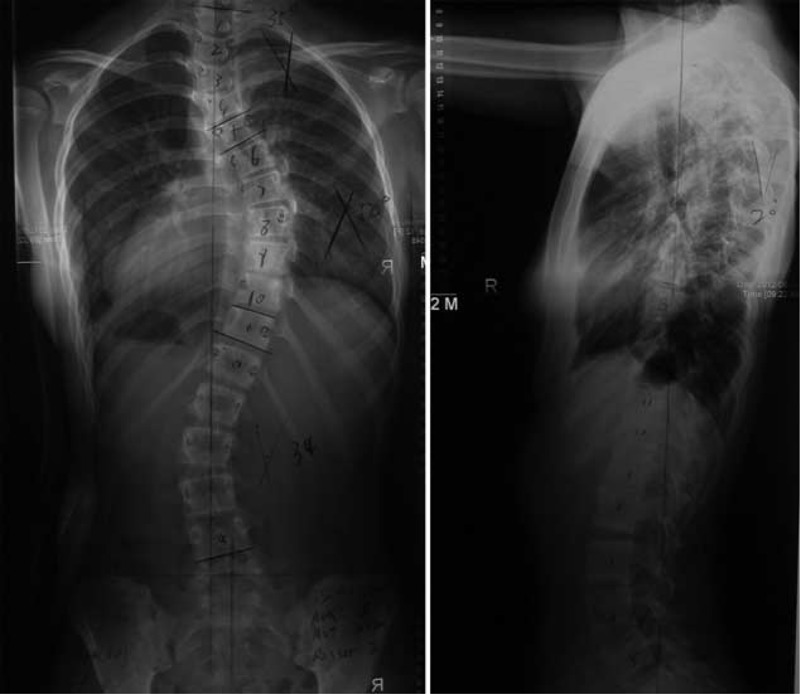
Standing anteroposterior and lateral radiographs of preoperation.

We present the case of a 12-year-old asylum seeker who was admitted for an elective correction of her progressive scoliotic deformity. Her plain radiographs of the spine showed that the thoracic scoliosis was progressive with the Cobb angles increasing from 36° to 56° (Figure 1), suggesting the need for surgical correction.

Her past medical history was only remarkable in that she had complained about a lot of menses amounts after 10 months of menarche. The patient denied any recent abdominal trauma, abdominopelvic pain, abnormal vaginal bleeding, nausea, vomiting, or diarrhea. She had undergone an ultrasound in a small medical clinic that revealed oblique vaginal septum and a didelphic uterus.

Ultrasounds in our hospital revealed a didelphic uterus and double cervix and absent right kidney. Atrial septal defect was also present while the cardiac function was normal. Magnetic resonance imaging (MRI) was further performed to evaluate other possible genitourinary anomaly. MRI imaging showed a uterine–vaginal anomaly consisting of didelphys uterus, double cervix, and double vagina, one of which was obstructed, and consequently, there was accumulation of fluid exhibiting a signal intensity similar to methemoglobin in the right uterus (slightly dilated, 14 mm diameter of the lumen) and in the right obstructed vagina. The diagnosis of HWWS was confirmed based on the points of didelphic uterus, unilateral renal absence, and hematometra.

**FIGURE 2:**
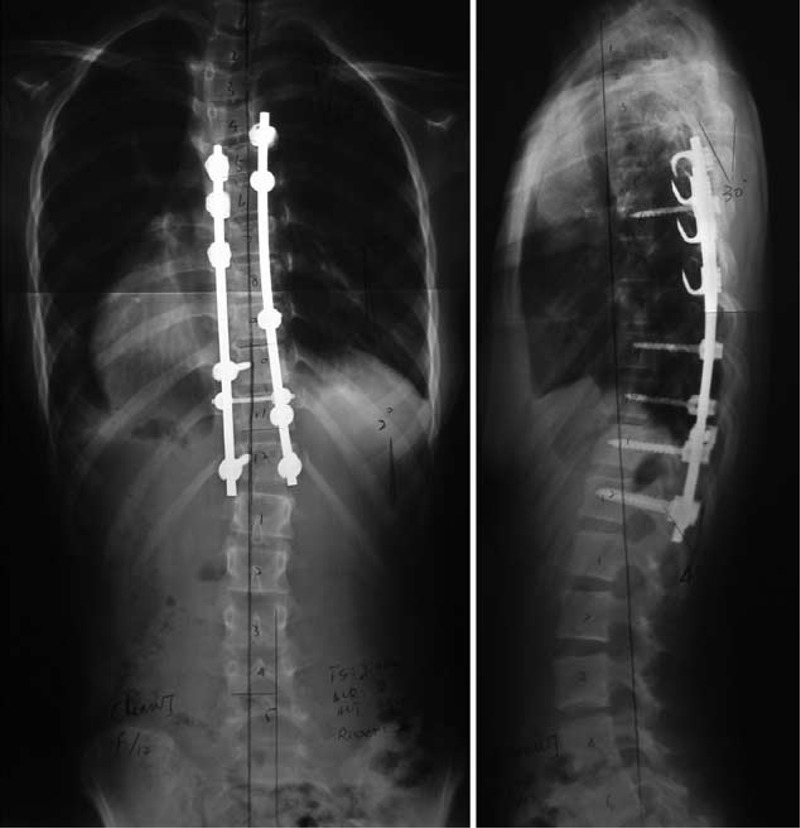
Standing anteroposterior and lateral radiographs of 4 days after operation.

**FIGURE 3:**
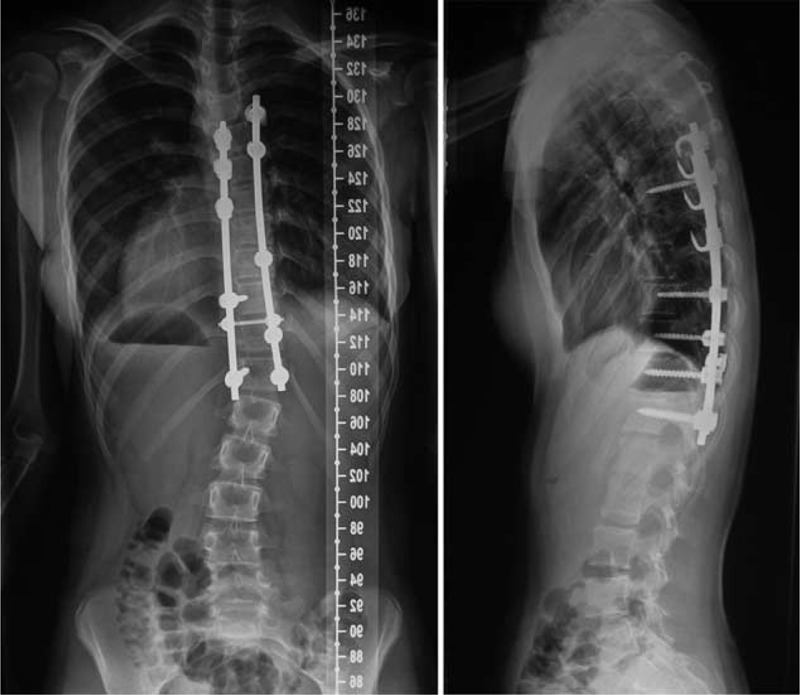
Standing anteroposterior and lateral radiographs of 24 months after reoperation.

In February 2012, a posterior correction and fusion at thoracic 5–thoracic 12 (T5–T12) levels was performed, using the Moss-SI spinal system. The total operation time was 3 hours and 15 minutes. Total amount of blood loss was 400 mL. During the operation, the signal of this patient was normal on intraoperative spinal cord monitoring. Postoperatively, there was no sign of renal dysfunction. Postoperative plain x-ray film demonstrated a Cobb angles correction from 54° to 12° (correction rate 76.9%) (Figure 2). Her follow-up was asymptomatic, well balanced in both the sagittal and coronal planes, with solid fusion (Figure 3) at the 24th postoperative month. Both patient and families were satisfied with the results of the surgery.

Six months after the scoliosis correction surgery, the patient went to our clinic for the treatment of HWWS. A vaginal septum resection was performed and the pyocolpos was drained. Both cervixes were also explored. After the operation, her symptom improved and she was discharged from the hospital on the second postoperative day. Her follow-up was symptom free at the fourth postoperative month.

To study the potential association between congenital scoliosis and HWWS, we evaluated the spinal deformity of the HWWS patients in our hospital between February 1997 and February 2014. All of them with the diagnosis of HWWS confirmed according to the clinical examination criteria. A total of 73 HWWS patients were identified. There were 6 cases of congenital scoliosis among those 73 HWWS patients. The prevalence of congenital scoliosis among patients with HWWS was 8.57%, which was much higher than the incidence of congenital scoliosis among general population (1/1000).

## DISCUSSION

The prevalence of HWWS worldwide was estimated to be 0.1% to 3.8%.^9,10^ In addition to the major clinical findings mentioned previously, there are limited reports regarding the diagnosis and management of HWWS with scoliosis. In the present study, we reported the case of a 12-year-old HWWS case with scoliosis. To our knowledge, this is the first report of scoliosis in the setting of HWWS.

Typically, HWWS syndrome presents with acute or chronic pelvic pain after the menarche.^7^ The retention of menstrual blood in the obstructed hemivagina leads to the formation of a hematocolpos, which is usually clinically detected as a pelvic mass after menarche.^11,12^ Diagnosis usually is delayed if a communication between the 2 vaginas exists.^5,13^ Both a dilated hemivagina and a dilated uterine cavity and fallopian tube may be present at the time of diagnosis.^5^ MRI and ultrasound are the most widely used diagnostic tools, with even 100% accuracy being reported for MRI because of its high accuracy and detailed elaboration of uterovaginal anatomy.^4,14^ Several studies have reported the renal malformations in HWWS, including renal agenesis, multicystic dysplastic kidney, and absent kidney. However, the scoliosis among HWWS has not been reported.^11,15,16^ There are no specific guidelines of scoliosis operations on patients with HWWS, but doctors must keep in mind that HWWS, including progressive pelvic pain and renal function impairment, may progress, possibly to abnormal before surgery.^10,17^

The exact etiology, pathogenesis, and embryologic origin of HWW syndrome are still not known.^7^ Wolffian ducts play an important role in the development of internal genital organs and kidneys.^2,14^ Incomplete or absent fusion of hemiuteri at eighth week of gestation results in the formation of 2 hemiuteri.^16,17^ Renal agenesis accompanies Müllerian duct abnormalities include the kidneys, fallopian tube, ovary, cervix, and upper vagina, all that generate from the same ureteric bud that fails to form correctly.^4,5^

The prevalence of congenital scoliosis among patients with HWWS was 8.57%, much higher than the incidence of congenital scoliosis among general population (1/1000). Both genetic variant and environment factors during pregnancy might play roles in the potential mechanisms in the pathogenesis of congenital scoliosis of HWWS. The main limitation for investigating the association between congenital scoliosis and HWWS is the sample size, as both of them are rare disease.

## CONCLUSION

In conclusion, HWWS is a relatively new rare syndrome described in recent years. Uterine didelphys, obstructed hemivagina, and ipsilateral renal agenesis are the most consistent features of the HWWS. There is a potential association between congenital scoliosis and HWWS. However, the exact association of all these conditions is unclear. As the number of cases increases, the etiology, clinical manifestations, and natural history of the syndrome will become clearer.
